# Supplementary material for: Long-term impact of molecular epidemiology shifts of methicillin-resistant Staphylococcus aureus on severity and mortality of bloodstream infection
Source: Emerg Microbes Infect. 2025 Jan 9;14(1):2449085. doi: 10.1080/22221751.2024.2449085 (PMC11727054; doi:10.1080/22221751.2024.2449085)
Supplement: Table S1.pdf [file TEMI_A_2449085_SM1482.pdf]

Supplementary Table 1.Changes in SCC*mec* types and patient characteristics from 2003 to 2019

	2003-2007 (n=83)		2008-2011 (n=83)		2012-2015 (n=27)		2016-2019 (n=58)		2003-2007 vs 2008-2011		2003-2007 vs 2012-2015		2003-2007 vs 2016-2019		2008-2011 vs 2012-2015		2008-2011 vs 2016-2019		2012-2015 vs 2016-2019	
	n	(%)	n	(%)	n	(%)	n	(%)	*P* value	*Q* value	*P* value	*Q* value	*P* value	*Q* value	*P* value	*Q* value	*P* value	*Q* value	*P* value	*Q* value
SCC*mec* types																				
SCC*mec* type I	2	(2.6%)	18	(23.1%)	10	(37.0%)	9	(15.5%)	<0.001	<0.001	<0.001	<0.001	0.009	0.019	n.s.		n.s.		0.048	0.072
SCC*mec* type II	61	(79.2%)	35	(44.9%)	10	(37.0%)	9	(15.5%)	<0.001	<0.001	<0.001	<0.001	<0.001	<0.001	n.s.		0.000	0.001	0.048	0.057
SCC*mec* type III	-	-	1	(1.3%)	-	-	-	-	n.s.		n.s.		n.s.		n.s.		n.s.		n.s.	
SCC*mec* type IV	14	(18.2%)	24	(30.8%)	7	(25.9%)	38	(65.5%)	0.092	0.139	n.s.		<0.001	<0.001	n.s.		<0.001	<0.001	0.001	0.002
SCC*mec* type IV	-	-	-	-	-	-	2	(3.4%)	n.s.		n.s.		0.183	n.s.	n.s.		0.180	n.s.	n.s.	
Patient characteristics																				
Age, average	63.7 ± 1.9		67.3 ± 1.6		69.1 ± 3.2		63.4 ± 3.2		n.s.		n.s.		n.s.		n.s.		n.s.		n.s.	
Sex, female	39	(47.0%)	25	(30.1%)	7	(25.9%)	21	(36.2%)	0.038	n.s.	n.s.		n.s.		n.s.		n.s.		n.s.	
Charlson comorbidity index	3.0 ± 0.2		3.1 ± 0.2		4.0 ± 0.5		3.2 ± 0.3		n.s.		n.s.		n.s.		n.s.		n.s.		n.s.	
Number of days of hospitalization after MRSA detection	No data		41.9 ± 6.3		38.5 ± 6.3		36.1 ± 7.9		-	-	-	-	-	-	n.s.	-	n.s.	-	n.s.	-
Antimicrobial use during the 30 days before MRSA detection	No data		63	(75.9%)	16	(59.3%)	35	(60.3%)	-	-	-	-	-	-	0.138	n.s.	0.063	0.189	n.s.	
Classification of infection																				
Community-acquired	No data		3	(3.6%)	0	(0.0%)	5	(8.6%)	-	-	-	-	-	-	n.s.		n.s.		0.173	n.s.
Healthcare-associated	No data		3	(3.6%)	6	(22.2%)	8	(13.8%)	-	-	-	-	-	-	0.007	0.020	0.051	0.076	n.s.	
Hospital-acquired	No data		72	(86.7%)	21	(77.8%)	45	(77.6%)	-	-	-	-	-	-	n.s.		0.176	n.s.	n.s.	
Source of MRSA infection																				
Intravascular device	15	(18.1%)	25	(30.1%)	8	(29.6%)	27	(46.6%)	0.102	n.s.	n.s.		<0.001	0.002	n.s.		0.053	0.158	0.162	0.195
Respiratory tract	14	(16.9%)	17	(20.5%)	3	(11.1%)	1	(1.7%)	n.s.		n.s.		0.004	0.013	n.s.		0.001	0.004	0.093	0.185
Skin/soft tissue or surgical site	9	(10.8%)	13	(15.7%)	7	(25.9%)	8	(13.8%)	n.s.		0.065	n.s.	n.s.		n.s.		n.s.		n.s.	
Abdomen	6	(7.2%)	4	(4.8%)	0	(0.0%)	5	(8.6%)	n.s.		n.s.		n.s.		n.s.		n.s.		n.s.	
Bone and joint	11	(13.3%)	2	(2.4%)	3	(11.1%)	5	(8.6%)	0.018	0.106	n.s.		n.s.		0.094	n.s.	0.124	n.s.	n.s.	
Others	6	(7.2%)	5	(6.0%)	3	(11.1%)	2	(3.4%)	n.s.		n.s.		n.s.		n.s.		n.s.		n.s.	
Unknown	22	(26.5%)	17	(20.5%)	3	(11.1%)	10	(17.2%)	n.s.		0.118	n.s.	n.s.		n.s.		n.s.		n.s.	
SOFA score	5.8 ± 0.5		5.8 ± 0.5		4.2 ± 0.9		3.1 ± 0.4		n.s.		n.s.		0.004		n.s.		0.005		n.s.	
Initial antimicrobial chemotherapy against MRSA																				
Vancomycin	24	(28.9%)	38	(45.8%)	15	(55.6%)	40	(69.0%)	0.037	0.055	0.020	0.039	<0.001	<0.001	n.s.		0.010	0.029	n.s.	
Teicoplanin	33	(39.8%)	11	(13.3%)	3	(11.1%)	3	(5.2%)	0.000	0.001	0.008	0.017	<0.001	<0.001	n.s.		0.155	n.s.	n.s.	
Linezolid	9	(10.8%)	18	(21.7%)	7	0.259	1	(1.7%)	0.091	0.109	0.065	0.098	0.047	0.094	n.s.		<0.001	0.002	0.001	0.003
Daptomycin	0	(0.0%)	0	(0.0%)	3	(11.1%)	12	(20.7%)	n.s		0.014	0.027	<0.001	<0.001	0.014	0.027	<0.001	<0.001	n.s.	
Arbekacin	3	(3.6%)	1	(1.2%)	0	(0.0%)	0	(0.0%)	n.s.		n.s.		n.s.		n.s.		n.s.		n.s.	
No anti-MRSA agents	14	(16.9%)	16	(19.3%)	0	(0.0%)	3	(5.2%)	n.s.		0.020	0.059	0.039	0.058	0.010	0.062	0.022	0.045	n.s.	
Change of initial treatment	29	(34.9%)	25	(30.1%)	7	(25.9%)	22	(37.9%)	n.s.		n.s.		n.s.		n.s.		n.s.		n.s.	
In-hospital mortality	33	(39.8%)	21	(25.3%)	10	(37.0%)	9	(15.5%)	0.068	0.136	n.s.		0.003	0.015	n.s.		n.s.		0.048	0.143
30-days mortality	No data		15	(18.1%)	5	(18.5%)	6	(10.3%)	-	-	-	-	-	-	n.s.		n.s.		n.s.	

n.s., *P* or *Q values* > 0.2
