# Supplementary figures and images for: Long-term impact of molecular epidemiology shifts of methicillin-resistant Staphylococcus aureus on severity and mortality of bloodstream infection
Source: Emerg Microbes Infect. 2025 Jan 9;14(1):2449085. doi: 10.1080/22221751.2024.2449085 (PMC11727054; doi:10.1080/22221751.2024.2449085)

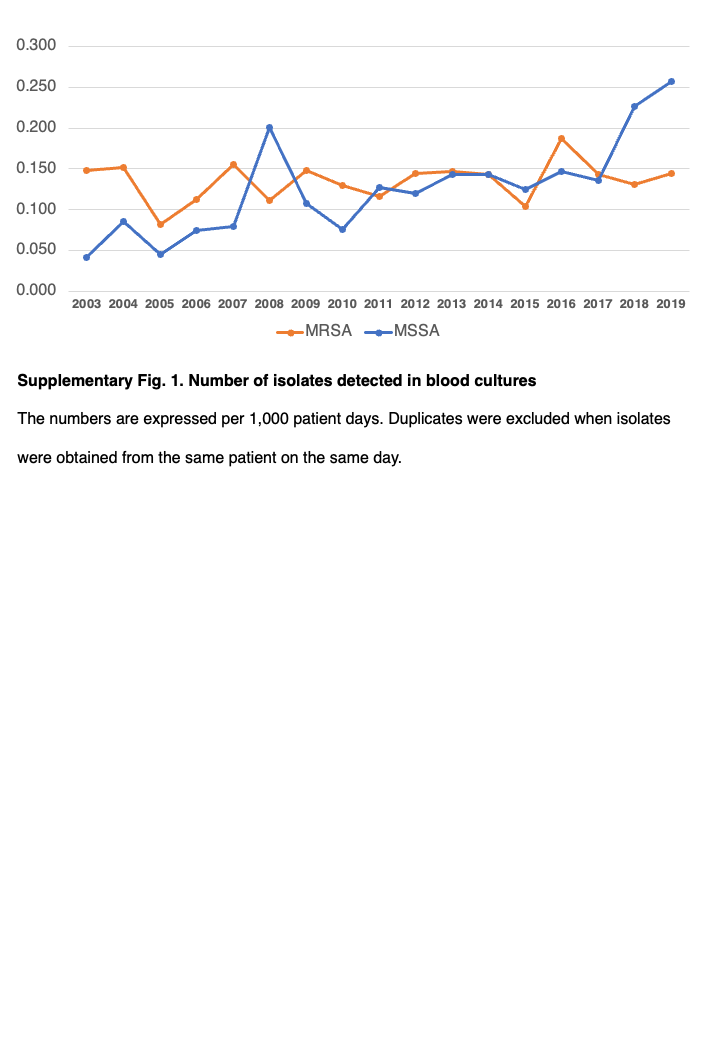

Supplement: Fig S1.tiff [file TEMI_A_2449085_SM1481.tiff]

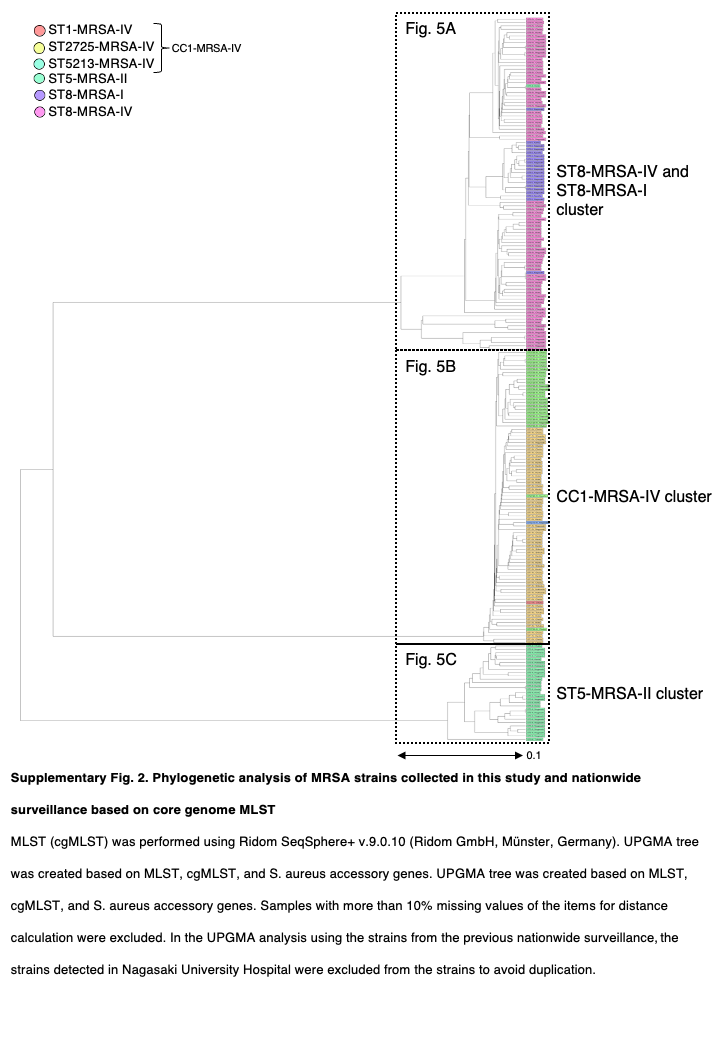

Supplement: Fig S2.tiff [file TEMI_A_2449085_SM1476.tiff]
