# Supplementary material for: Long-term impact of molecular epidemiology shifts of methicillin-resistant Staphylococcus aureus on severity and mortality of bloodstream infection
Source: Emerg Microbes Infect. 2025 Jan 9;14(1):2449085. doi: 10.1080/22221751.2024.2449085 (PMC11727054; doi:10.1080/22221751.2024.2449085)
Supplement: Table S5.pdf [file TEMI_A_2449085_SM1480.pdf]

Supplementary Table 5.Changes in outcomes from 2003 to 2019 with the same inclusion criteria

	2003-2007 (n=83)		2008-2011 (n=83)		2012-2015 (n=70)		2016-2019 (n=94)		2003-2007 vs 2008-2011		2003-2007 vs 2012-2015		2003-2007 vs 2016-2019		2008-2011 vs 2012-2015		2008-2011 vs 2016-2019		2012-2015 vs 2016-2019	
	n	(%)	n	(%)	n	(%)	n	(%)	*P* value	*Q* value	*P* value	*Q* value	*P* value	*Q* value	*P* value	*Q* value	*P* value	*Q* value	*P* value	*Q* value
In-hospital mortality	33	(39.8%)	21	(25.3%)	17	(24.3%)	16	(17.0%)	0.068	0.136	0.057	0.170	0.001	0.008	n.s.		0.198	n.s.	n.s.	
30-days mortality	No data		15	(18.1%)	5	(15.7%)	10	(10.6%)	-	-	-	-	-	-	n.s.		n.s.		n.s.	

n.s., *P* or *Q values* > 0.2
