# Supplementary material for: Long-term impact of molecular epidemiology shifts of methicillin-resistant Staphylococcus aureus on severity and mortality of bloodstream infection
Source: Emerg Microbes Infect. 2025 Jan 9;14(1):2449085. doi: 10.1080/22221751.2024.2449085 (PMC11727054; doi:10.1080/22221751.2024.2449085)
Supplement: Table S4.pdf [file TEMI_A_2449085_SM1478.pdf]

Supplementary Table 4. Comparison of patient and strain characteristics according to the sug-groups in ST8-IV

	CA-MRSA/J (n=13)	t5071-ST8-IV (n=4)	*p* value
Patient characteristics			
Age, average	75.2 ± 3.2	58.8 ± 19.4	0.175
Sex, female	5 (38.5%)	2 (50.0%)	n.s.
Charlson comorbidity index	3.8 ± 0.8	2.0 ± 0.7	n.s.
Number of days of hospitalization after MRSA detection	38.1 ± 11.1	27.5 ± 27.5	n.s.
Antimicrobial use during the 30 days before MRSA detection	7 (53.8%)	1 (25.0%)	n.s.
Classification of infection			
Community-acquired	0 (0.0%)	1 (25.0%)	n.s.
Healthcare-associated	2 (15.4%)	2 (50.0%)	n.s.
Hospital-acquired	11 (84.6%)	1 (25.0%)	0.053
Source of MRSA infection			
Intravascular device	5 (38.5%)	1 (25.0%)	n.s.
Skin/soft tissue or surgical site	3 (23.1%)	1 (25.0%)	n.s.
Abdomen	1 (7.7%)	0 (0.0%)	n.s.
Bone and joint	0 (0.0%)	0 (0.0%)	n.s.
Others	1 (7.7%)	0 (0.0%)	n.s.
Unknown	3 (23.1%)	2 (50.0%)	n.s.
SOFA score	4.6 ± 1.3	1.0 ± 0.0	0.166
Initial antimicrobial chemotherapy against MRSA			
Vancomycin	10 (76.9%)	3 (75.0%)	n.s.
Teicoplanin	1 (7.7%)	0 (0.0%)	n.s.
Linezolid	1 (7.7%)	1 (25.0%)	n.s.
Daptomycin	1 (7.7%)	0 (0.0%)	n.s.
No anti-MRSA agents	0 (0.0%)	0 (0.0%)	n.s.
Change of initial treatment	7 (53.8%)	1 (25.0%)	n.s.
In-hospital mortality	3 (23.1%)	1 (25.0%)	n.s.
30-days mortality	3 (23.1%)	1 (25.0%)	n.s.
Strain characteristics			
Drug resistance rate according to CLSI			
Oxacillin	13 (100%)	4 (100%)	n.s.
Cefoxitin	13 (100%)	4 (100%)	n.s.
Levofloxacin	0 (0.0%)	4 (100%)	<0.001
Erythromycin	6 (46.2%)	4 (100%)	0.103
Clindamycin	1 (7.7%)	3 (75.0%)	0.022
Minocycline	0 (0.0%)	0 (0.0%)	n.s.
Vancomycin	0 (0.0%)	0 (0.0%)	n.s.
Teicoplanin	0 (0.0%)	0 (0.0%)	n.s.
Linezolid	0 (0.0%)	0 (0.0%)	n.s.
Drug resistance rate according to EUCAST			
Oxacillin	13 (100%)	4 (100%)	n.s.
Cefoxitin	13 (100%)	4 (100%)	n.s.
Levofloxacin	0 (0.0%)	4 (100%)	<0.001
Erythromycin	6 (46.2%)	4 (100%)	0.103
Clindamycin	1 (7.7%)	4 (100%)	0.002
Minocycline	0 (0.0%)	2 (50.0%)	0.044
Vancomycin	0 (0.0%)	0 (0.0%)	n.s.
Teicoplanin	0 (0.0%)	0 (0.0%)	n.s.
Linezolid	0 (0.0%)	0 (0.0%)	n.s.
spa type			
t8	1 (7.7%)	0 (0.0%)	n.s.
t24	0 (0.0%)	0 (0.0%)	n.s.
t351	0 (0.0%)	0 (0.0%)	n.s.
t1767	5 (38.5%)	0 (0.0%)	n.s.
t2083	1 (7.7%)	0 (0.0%)	n.s.
t2229	1 (7.7%)	0 (0.0%)	n.s.
t3286	0 (0.0%)	0 (0.0%)	n.s.
t5071	0 (0.0%)	4 (100%)	<0.001
t6127	0 (0.0%)	0 (0.0%)	n.s.
t16888	0 (0.0%)	0 (0.0%)	n.s.
Unknown	5 (38.5%)	0 (0.0%)	n.s.
Aminoglycoside-resistance genes	13 (100%)	4 (100%)	n.s.
aac(6')-aph(2'')	13 (100%)	0 (0.0%)	<0.001
aadD	11 (84.6%)	0 (0.0%)	<0.001
ant(9)-Ia	2 (15.4%)	4 (100%)	0.006
Beta-lactamase, blaZ	11 (84.6%)	4 (100%)	n.s.
Chloramphenicol-resistance genes, *cat(pC221)*	0 (0.0%)	1 (25.0%)	n.s.
Fosmycin-resistance	0 (0.0%)	0 (0.0%)	n.s.
fosB6	0 (0.0%)	0 (0.0%)	n.s.
fosD	0 (0.0%)	0 (0.0%)	n.s.
Macrolide-resistance	7 (53.8%)	4 (100%)	n.s.
erm(A)	2 (15.4%)	4 (100%)	0.006
erm(C)	5 (38.5%)	0 (0.0%)	n.s.
msr(A)	0 (0.0%)	0 (0.0%)	n.s.
Tetracycline-resistance	2 (15.4%)	2 (50.0%)	n.s.
Tet (K)	1 (7.7%)	0 (0.0%)	n.s.
Tet (M)	1 (7.7%)	2 (50.0%)	0.121
Bleomycin-resistance, *bleO*	11 (84.6%)	0 (0.0%)	<0.001
Exoenzyme genes			
aur	13 (100%)	4 (100%)	n.s.
splA	13 (100%)	4 (100%)	n.s.
splB	13 (100%)	4 (100%)	n.s.
splE	0 (0.0%)	3 (75.0%)	0.006
Toxin genes			
edinA	6 (46.2%)	0 (0.0%)	n.s.
eta	0 (0.0%)	0 (0.0%)	n.s.
hlgA	13 (100%)	4 (100%)	n.s.
hlgB	13 (100%)	4 (100%)	n.s.
hlgC	13 (100%)	4 (100%)	n.s.
LukD	13 (100%)	4 (100%)	n.s.
LukE	13 (100%)	4 (100%)	n.s.
LukF-PV	0 (0.0%)	0 (0.0%)	n.s.
sea	0 (0.0%)	0 (0.0%)	n.s.
seb	0 (0.0%)	0 (0.0%)	n.s.
sec	13 (100%)	0 (0.0%)	<0.001
sec3	0 (0.0%)	0 (0.0%)	n.s.
sed	1 (7.7%)	0 (0.0%)	n.s.
seg	0 (0.0%)	0 (0.0%)	n.s.
seh	0 (0.0%)	0 (0.0%)	n.s.
sei	0 (0.0%)	0 (0.0%)	n.s.
sej	1 (7.7%)	0 (0.0%)	n.s.
sek	0 (0.0%)	0 (0.0%)	n.s.
sel	12 (92.3%)	0 (0.0%)	<0.001
sem	0 (0.0%)	0 (0.0%)	n.s.
sen	0 (0.0%)	0 (0.0%)	n.s.
seo	0 (0.0%)	0 (0.0%)	n.s.
sep	3 (23.1%)	4 (100%)	0.015
seq	0 (0.0%)	0 (0.0%)	n.s.
ser	1 (7.7%)	0 (0.0%)	n.s.
seu	0 (0.0%)	0 (0.0%)	n.s.
tst	12 (92.3%)	0 (0.0%)	0.002
Others			
ACME	0 (0.0%)	0 (0.0%)	n.s.
sak	12 (92.3%)	4 (100%)	n.s.
scn	12 (92.3%)	4 (100%)	n.s.

n.s., *P* or *Q values* > 0.2
