# Supplementary material for: Long-term impact of molecular epidemiology shifts of methicillin-resistant Staphylococcus aureus on severity and mortality of bloodstream infection
Source: Emerg Microbes Infect. 2025 Jan 9;14(1):2449085. doi: 10.1080/22221751.2024.2449085 (PMC11727054; doi:10.1080/22221751.2024.2449085)
Supplement: Table S2.pdf [file TEMI_A_2449085_SM1477.pdf]

Supplementary Table 2. Combination of ST and SCC*mec* type in 2012-2019

	All (n=85)		2012-2015 (n=27)		2016-2019 (n=58)		*P* value 2012-2015 vs 2016-2019
	n	(%)	n	(%)	n	(%)	
CC1-MRSA-IV	8	(9.4%)	-	-	8	(13.8%)	0.051
ST1-MRSA-IV	3	(3.5%)	-	-	3	(5.2%)	n.s.
ST2725-MRSA-IV	4	(4.7%)	-	-	4	(6.9%)	n.s.
ST5213-MRSA-IV	1	(1.2%)	-	-	1	(1.7%)	n.s.
CC5-MRSA-II	17	(20.0%)	9	(33.3%)	8	(13.8%)	0.045
ST5-MRSA-II	16	(18.8%)	8	(29.6%)	8	(13.8%)	0.134
ST764-MRSA-II	1	(1.2%)	1	(3.7%)	-	-	n.s.
CC5-MRSA-IV	3	(3.5%)	1	(3.7%)	2	(3.4%)	n.s.
ST5-MRSA-IV	3	(3.5%)	1	(3.7%)	2	(3.4%)	n.s.
CC8-MRSA-I	19	(22.4%)	10	(37.0%)	9	(15.5%)	0.048
ST8-MRSA-I	19	(22.4%)	10	(37.0%)	9	(15.5%)	0.048
CC8-MRSA-II	2	(2.4%)	1	(3.7%)	1	(1.7%)	n.s.
ST8-MRSA-II	2	(2.4%)	1	(3.7%)	1	(1.7%)	n.s.
CC8-MRSA-IV	34	(40.0%)	6	(22.2%)	28	(48.3%)	0.032
ST8-MRSA-IV	32	(37.6%)	6	(22.2%)	26	(44.8%)	0.056
ST2516-MRSA-IV	1	(1.2%)	-	-	1	(1.7%)	n.s.
ST8465-MRSA-IV	1	(1.2%)	-	-	1	(1.7%)	n.s.
CC121-MRSA-V	2	(2.4%)	-	-	2	(3.4%)	n.s.
ST121-MRSA-V	2	(2.4%)	-	-	2	(3.4%)	n.s.

n.s., $p > 0.2$
